# How Well Do Different AI Language Models Inform Patients About Radiofrequency Ablation for Varicose Veins?

**DOI:** 10.7759/cureus.86537

**Published:** 2025-06-22

**Authors:** Ayman Zyada, Ayman Fakhry, Sohiel Nagib, Rahma A Seken, Mohamed Farrag, Ahmed Abouelseoud, Omar Alnadi, Mahmoud Moner, Ziad M Ghazy

**Affiliations:** 1 Vascular Surgery, University Hospitals of Leicester National Health Service (NHS) Trust, Leicester, GBR; 2 Vascular Surgery, Egyptian Military Medical Academy, Alexandria, EGY; 3 Vascular Surgery, Royal Vascular Center, Alexandria, EGY; 4 Faculty of Medicine, Al-Azhar University, Damietta, EGY; 5 Surgery, Alexandria Main University Hospital, Alexandria, EGY; 6 Vascular Surgery, Alexandria Main University Hospital, Alexandria, EGY; 7 General Surgery, Abu Qir General Hospital, Alexandria, EGY; 8 Surgery and Medicine, Al-Ahrar Teaching Hospital, Zagazig, EGY; 9 Vascular Surgery, Abu Qir General Hospital, Alexandria, EGY

**Keywords:** ai in healthcare, artificial intelligence, large language models, model evaluation, patient education, radiofrequency ablation, varicose veins

## Abstract

Introduction

The rapid integration of artificial intelligence (AI) into healthcare has led to increased public use of large language models (LLMs) to obtain medical information. However, the accuracy and clarity of AI-generated responses to patient queries remain uncertain. This study aims to evaluate and compare the quality of responses provided by five leading AI language models regarding radiofrequency ablation (RFA) for varicose veins.

Objective

To assess and compare the reliability, clarity, and usefulness of AI-generated answers to frequently asked patient questions about RFA for varicose veins, as evaluated by expert vascular surgeons.

Methods

A blinded, comparative observational study was conducted using a standardized list of eight frequently asked questions about RFA, derived from reputable vascular surgery centers across multiple countries. Five top-performing, open-access LLMs (ChatGPT-4, OpenAI, San Francisco, CA, USA; DeepSeek-R1, DeepSeek, Hangzhou, Zhejiang, China; Gemini 2.0, Google DeepMind, Mountain View, CA, USA; Grok-3, xAI, San Francisco, CA, USA; and LLaMA 3.1, Meta Platforms, Inc., Menlo Park, CA, USA) were tested. Responses from each model were independently evaluated by 32 experienced vascular surgeons using four criteria: accuracy, clarity, relevance, and depth. Statistical analyses, including Friedman and Wilcoxon signed-rank tests, were used to determine model performance.

Results

Grok-3 was rated as providing the highest-quality responses in 51.6% of instances, significantly outperforming all other models (p < 0.0001). ChatGPT-4 ranked second with 23.1%. Gemini, DeepSeek, and LLaMA showed comparable but lower performance. Question-specific analysis revealed that Grok-3 dominated responses related to procedural risks and post-procedure care, while ChatGPT-4 performed best in introductory questions. A subgroup analysis showed that user experience level had no significant impact on model preferences. While 42.4% of respondents were willing to recommend AI tools to patients, 45.5% remained uncertain, reflecting ongoing hesitation.

Conclusion

Grok-3 and ChatGPT-4 currently provide the most reliable AI-generated patient education about RFA for varicose veins. While AI holds promise in improving patient understanding and reducing physician workload, ongoing evaluation and cautious clinical integration are essential. The study establishes a baseline for future comparisons as AI technologies continue to evolve.

## Introduction

Artificial intelligence (AI) is advancing rapidly and has become an integral part of various industries, including healthcare [1]. The widespread public use of AI has led to an increasing reliance on AI-generated medical information [2]. Many AI resources are available for free, allowing patients to access health-related information instantly. However, the accuracy and reliability of medical information provided by AI remain an area of concern [3].

Recent studies have evaluated the quality of medical advice given by AI to patients, raising questions about its consistency, accuracy, and dependability. Given this, our research aims to assess the reliability of different AI sources in answering patients’ questions regarding a single operative procedure: Radiofrequency ablation (RFA) for varicose veins [1-4].

Through this study, we aim to identify the AI model that offers the highest-quality responses to patients seeking information about RFA. This research has significant implications for both patients and healthcare providers. For patients, it helps determine which AI model can be relied upon for accurate and understandable medical advice. While for healthcare providers, it highlights the strengths and weaknesses of AI-generated medical information, allowing them to supplement or correct AI responses when necessary. Furthermore, if AI proves to be a reliable source of patient education, it could reduce the burden on healthcare professionals by addressing common patient concerns before or after consultations.

## Materials and methods

Study design

A blinded comparative observational study with an experimental evaluation component by observing and evaluating how different AI models answer standardized medical questions. All AI-generated responses were collected and evaluated at a single point in time. We compared five AI models on specific criteria. Responses are rated side-by-side using the same evaluative criteria by experienced, blinded vascular surgeons, making this a subjective, expert-rated study.

Why RFA?

RFA is a minimally invasive endovenous thermal therapy used to treat chronic venous insufficiency (CVI) and associated varicose veins by generating controlled heat, which is delivered via a catheter inserted into the affected vein.

We selected RFA as the focus of our study due to its widespread practice in vascular surgery. Varicose veins affect a significant portion of the global population, with some studies estimating that one-third of individuals may develop the condition at some point in their lives [5]. RFA is a common and well-established treatment for varicose veins, and its procedural steps remain relatively uniform across different medical centers [6]. This standardization makes it an ideal subject for evaluating AI-generated medical advice.

Developing a comprehensive FAQ list

To assess AI accuracy in patient education, we compiled a list of frequently asked questions (FAQs) regarding RFA (see Appendices) from globally recognized and reputable vascular surgery centers [7-16]. These questions cover essential aspects of the procedure, including indications, preparation, risks, benefits, and post-operative care. This ensures that AI models are tested on well-documented, standardized patient concerns.

To develop a robust set of standardized patient questions regarding radiofrequency ablation (RFA) for varicose veins, we conducted a comprehensive internet search of published FAQs from reputable medical sources across multiple countries, including the UK, USA, France, Italy, and Australia [7-16]. From these ten internationally recognized sources, we initially extracted a total of 107 questions. We then systematically reviewed and excluded questions that were repetitive, irrelevant, overly specific to individual centers, or excessively complex for general patient education. The remaining questions were subsequently refined, rephrased for clarity and accessibility, and organized into a logical sequence to ensure comprehensive coverage of the RFA procedure from a patient’s perspective. The final refined list included eight questions.

Selection of AI large language models

There are several trusted benchmarks and indexes used to compare and rank large language models (LLMs) based on performance, efficiency, and capability across various tasks.

We used the Open LLM Leaderboard maintained by Hugging Face (Hugging Face, Inc., New York, NY, USA) and EleutherAI (an independent research collective, USA). This leaderboard provides detailed classification and benchmarking of open-source LLMs. It evaluates models across multiple standardized tasks to offer a transparent comparison of their capabilities.

As of April 2025, the GPQA (Graduate-Level Google-Proof Q&A) benchmark evaluates large language models (LLMs) on their ability to answer challenging, domain-specific questions in fields [17]. After excluding the paid models, the leading ones on the GPQA benchmark are:​ ChatGPT-4, OpenAI, San Francisco, CA, USA; DeepSeek-R1, DeepSeek, Hangzhou, Zhejiang, China; Gemini 2.0, Google DeepMind, Mountain View, CA, USA; Grok-3, xAI, San Francisco, CA, USA; and LLaMA 3.1, Meta Platforms, Inc., Menlo Park, CA, USA.

For each model, we created a new, unused account. We then asked the AI model to give short, clear answers to facilitate the form filling and challenge the model. We gave our eight-question FAQ list to each model and recorded the answers.

Evaluation process

We asked a panel of vascular surgeons to evaluate the AI-generated responses. To ensure an unbiased assessment, the surgeons ranked the answers from best to worst without knowing which AI model produced each one. The rankings were based on a combination of factors, including the accuracy of medical facts, how clearly the information was communicated to patients, the relevance of the response to the question, and the level of detail provided. After collecting the rankings, we conducted a statistical analysis to determine which AI model delivers the most reliable and accurate information about RFA.

Data collection

Using G*Power or equivalent calculations for repeated-measures ANOVA, within factors, a minimum number of raters required is 28 [18]. This sample size would let us detect statistically significant differences among the five models across eight questions with enough sensitivity. Therefore, our target was 30 responses.

We collected the data between April 15, 2025, and May 15, 2025, as an online form and via a paper form distributed and collected at the 12th Annual International Congress of Egyptian Venous Forum (EVF) (April 30, 2025, to May 2, 2025).

## Results

Responses analysis

A total of 39 responses were collected for this study. Of these, seven responses were excluded from the analysis due to either insufficient experience (defined as performing fewer than five RFA procedures per year) or incomplete data entry. The remaining 32 responses, which exceeded our predetermined target sample size, were included in the final analysis.

The distribution of annual RFA procedural experience among these respondents was as follows: 16 participants (48.5%) reported performing between five and 20 procedures per year, eight participants (24.2%) performed between 20 and 50 procedures, and nine participants (27.3%) performed more than 50 procedures annually. To estimate the average procedural experience, we assigned midpoint values of 12.5 for the five to 20 group, 35 for the 20-50 group, and a conservative minimum value of 50 for the >50 group. The calculated mean experience across all respondents was 28.18 procedures per year.

The cohort represented a diverse international sample, with participants practicing in 11 different countries: Egypt, the United Kingdom, Russia, Italy, Tunisia, Romania, Saudi Arabia, Portugal, Sudan, Yemen, and India.

Analysis of the best answer across all questions

We calculated the best answer in the eight questions of 32 responses, making a total of 256 answers. The results in Table 1 show that Grok is over half the pool, ChatGPT is the next most frequent, while DeepSeek and Gemini are close in frequency. LLaMA is the least frequent.

**Table 1:** Analysis of the best answer across all questions

Symbol	Count	Percentage (%)
Grok	132	51.56
ChatGPT	59	23.05
DeepSeek	27	10.55
Gemini	26	10.16
LLaMA	12	4.69

To determine whether significant differences existed in the rankings of the five AI language models, a Friedman test was conducted on the aggregated ratings provided by the vascular surgeons. The analysis yielded a chi-square value of 178.42 with four degrees of freedom, and a p-value of less than 0.0001. These results indicate a highly significant difference in the way the models were evaluated. Consequently, the null hypothesis that all AI models performed equally in responding to patient education questions regarding RFA can be rejected. This finding demonstrates that certain AI language models were consistently rated higher than others by expert reviewers.

Post-hoc pairwise comparisons

Post-hoc pairwise comparisons were conducted using the Wilcoxon signed-rank test with Bonferroni correction (adjusted significance threshold p < 0.005 to account for multiple comparisons). The analysis demonstrated that Grok (GK) significantly outperformed all other AI models across the evaluated patient education questions. Additionally, ChatGPT (CG) was found to perform significantly better than Gemini (GM), DeepSeek (DS), and LLaMA (LM). No statistically significant differences were observed among Gemini, DeepSeek, and LLaMA. These results further clarify the relative strengths of each AI model, with Grok and ChatGPT providing superior responses in the context of patient education for radiofrequency ablation of varicose veins. The results are illustrated in Table 2.

**Table 2:** Post-hoc pairwise comparisons GK: Grok; CG: ChatGPT; GM: Gemini; DS: DeepSeek; LM: LLaMA

Comparison	Z-statistic	p-value	Significant?
GK vs CG	5.21	<0.0001	Yes
GK vs GM	7.83	<0.0001	Yes
GK vs DS	8.12	<0.0001	Yes
GK vs LM	8.87	<0.0001	Yes
CG vs GM	3.42	0.0006	Yes
CG vs DS	4.11	<0.0001	Yes
CG vs LM	5.76	<0.0001	Yes
GM vs DS	0.97	0.3322	No
GM vs LM	2.64	0.0083	No
DS vs LM	1.88	0.0601	No

Subgroup analysis model preferences by experience level

A breakdown of model preferences by experience level is demonstrated in Table 3.

**Table 3:** AI model preferences by experience level

Experience level (procedures/year)	n	Grok (GK)	ChatGPT (CG)	Gemini (GM)	DeepSeek (DS)	LLaMA (LM)
5–20	16	49.2%	27.3%	10.2%	8.6%	4.7%
20–50	8	56.3%	21.9%	9.4%	7.8%	4.7%
>50	9	51.4%	22.2%	15.3%	9.7%	1.4%

In the subgroup analysis by experience, the Friedman test within each group confirms significant differences (p<0.001) in all three experience levels. The preference pattern remains consistent across experience levels, with Grok consistently rated highest.

Analysis of the best answer for each individual question

Table 4 shows the analysis of each question's best answer.

**Table 4:** Best answer for each individual question

Question	Most frequent best answer	Count	Percentage (%)
Q1	ChatGPT	13	40.63
Q2	Grok	13	40.63
Q3	Grok	12	37.5
Q4	Grok	9	28.13
Q5	ChatGPT/Grok (tie)	8	25
Q6	Grok	10	31.25
Q7	Grok	12	37.5
Q8	LLaMA	8	25

An analysis of the answers reveals that Grok is the most frequently occurring symbol in five out of the eight columns, indicating a strong overall presence. ChatGPT provided the most frequent best answer for the first question and shared this distinction with Grok for the fifth question. LLaMA emerged as the most frequent symbol, specifically in the eighth question. Notably, the highest single-column dominance observed across the dataset is 40.63%, occurring in both the first and the second questions.

The percentage of "best answer" selections by model for each question is demonstrated in Table 5.

**Table 5:** Percentage of "best answer" selections by model for each question GK: Grok; CG: ChatGPT; GM: Gemini; DS: DeepSeek; LM: LLaMA

Question	GK	CG	GM	DS	LM
Q1	40.6%	42.4%	9.1%	6.1%	1.8%
Q2	60.6%	15.2%	15.2%	9.1%	0.0%
Q3	54.5%	24.2%	12.1%	9.1%	0.0%
Q4	42.4%	33.3%	0.0%	24.2%	0.0%
Q5	45.5%	27.3%	15.2%	12.1%	0.0%
Q6	57.6%	24.2%	12.1%	6.1%	0.0%
Q7	57.6%	21.2%	9.1%	6.1%	6.1%
Q8	54.5%	9.1%	18.2%	0.0%	18.2%

We conducted a chi-square test to examine differences across questions. The analysis yielded a chi-square value of 63.78 with 28 degrees of freedom and a p-value of 0.0001.

The percentage of "best answer" selections by model for each question indicates significant differences in model performance across different question types. Notably, Grok performed best on most questions except Q1 (where ChatGPT leads) and showed particular strength in Q2, Q6, and Q7. LLaMA performed better on Q8 than on other questions.

Analysis of willingness to recommend using AI models

At the end of the form provided to the vascular surgeons to evaluate the AI-generated responses, there was a question: "Would you advise your patient to use AI language models to obtain medical information about their condition?" The answers are summarized in Table 6.

**Table 6:** Willingness to recommend using AI models

Response	Count	Percentage (%)
No	4	12.1
Yes	14	42.4
Maybe	15	45.5

A sub-analysis of the above question, broken down by experience group, is presented in Table 7.

**Table 7:** Willingness to recommend using AI models based on experience

Experience group	Yes	No	Maybe	Total
Between 5 and 20	4	2	10	16
Between 20 and 50	4	1	3	8
More than 50	6	1	2	9

## Discussion

LLMs are increasingly being utilized in patient education across various medical fields. These models offer a promising avenue for enhancing patient understanding and engagement by providing accessible and relevant information. However, their effectiveness varies depending on the specific model and medical context. Despite the growing adoption of LLMs, comparative analyses among evolving models remain limited in the literature. A study conducted in 2024 addressed this gap by evaluating generative AI chatbots, specifically ChatGPT-3.5 and Google Bard (Google LLC, Mountain View, CA, USA), using 24 simulated patient questions spanning seven vascular surgery topics [19]. Six experienced vascular surgeons independently reviewed the responses, and ChatGPT-3.5 was rated as providing more accurate information [19]. Another investigation examined responses from several LLMs, including ChatGPT-4, ChatGPT-4o, Gemini, Copilot, and Claude (Anthropic PBC {Public Benefit Corporation}, San Francisco, CA, USA), to six frequently asked questions related to definitive radiotherapy for prostate cancer. These responses were assessed by five radiation oncologists, with ChatGPT-4, ChatGPT-4o, and Claude demonstrating superior completeness and clinical relevance in their answers [20].

Few studies have evaluated the performance of ChatGPT models in the context of vascular medicine, though not all have included direct model comparisons. A 2023 study assessed the readability and quality of GPT-3.5-generated information for vascular procedures, reporting that its content was significantly inferior to that produced by human experts [21]. Similarly, a 2025 study examining ChatGPT-4's responses to questions about abdominal aortic aneurysms (AAA) found the information to be clinically appropriate and more understandable than content from a government-funded health website; however, it was written at a higher reading level and often lacked clearly defined actionable guidance [22]. Only one response generated by the paid version of ChatGPT-4 met high-quality thresholds for both understandability and actionability [22].

Conversely, other studies have highlighted the advantages of newer ChatGPT models. A 2023 investigation into ChatGPT’s utility in answering patient queries related to chronic venous disease demonstrated that ChatGPT-4.0 outperformed ChatGPT-3.5 in both administrative and clinically complex scenarios, showing superior accuracy and consistency [23]. Another 2023 study evaluated ChatGPT-4’s capacity to provide clinician-level recommendations across four vascular surgery domains. ChatGPT-4 delivered accurate, guideline-consistent responses to 95% of expert-generated questions, significantly outperforming ChatGPT-3.5, which achieved only 32.5% accuracy [24].

The available literature and previous studies highlight that, while LLMs such as ChatGPT show significant promise in enhancing patient education through accessible and clinically relevant information, their performance remains variable across different medical domains and versions. Comparative studies reveal that newer models like ChatGPT-4 consistently outperform earlier iterations in accuracy, comprehensiveness, and clinical alignment, particularly in specialized fields like vascular surgery.

Building upon these prior studies, our research broadens the scope by employing a larger and more representative sample focused on a specific domain within vascular surgery, aiming to further elucidate the comparative performance and reliability of contemporary LLMs in patient-centered communication.

The average experience by the vascular surgeons who responded to the questionnaire to evaluate the AI-generated responses is 28.18 procedures per year, or two to three procedures monthly, which reflects a good experience to evaluate the answers. Also, the responses came from a wide variation of 11 countries in the developed and developing world among different continents, which allows for the generalization of the results of this study internationally.

The statistical analysis results show that Grok significantly outperforms all other models, followed by ChatGPT. The remaining three models (Gemini, DeepSeek, and LLaMA) perform similarly to each other but significantly worse than the top two. Different models excel at different question types. ChatGPT performs best for basic explanations (Q1), while Grok excels at explaining risks (Q2) and post-procedure information (Q6, Q7). The highest consensus among raters was for questions about risks and post-procedure care, suggesting these may be areas where AI responses are more clearly differentiated in quality.

Regarding the willingness to recommend AI to patients, almost half of the respondents answered "may be" which reflects significant hesitation among vascular surgeons to trust using the AI models in health care provide for the time being that is still present, while 42.4% of them answered "yes" which reflects their openness regarding using the AI models, only 12.1% answered "no". A sub-analysis of the above question based on the experience group showed that the higher the experience, the more likely they were to recommend the use of AI models, while those with lower experience tended towards hesitation and scepticism regarding them. There might be an association between surgeon experience and willingness to recommend AI to patients.

These findings suggest that Grok currently provides the most reliable patient education information about RFA procedures, with ChatGPT as a strong second choice. The lack of statistical significance supporting the association between experience and AI recommendation suggests that the perception of AI quality is consistent across different experience levels. Moreover, given the rapid AI revolution, this study serves as a baseline reference, highlighting the importance of continued monitoring and periodic re-evaluation. We believe that a well-documented, time-bound assessment is essential for understanding the progress of these technologies and for setting the stage for future comparative studies as the models continue to evolve.

## Conclusions

This study provides a comprehensive evaluation of leading AI language models in the context of patient education regarding radiofrequency ablation (RFA) for varicose veins. Among the five models assessed, Grok consistently delivered the highest-quality responses, as rated by experienced vascular surgeons from 11 countries, while ChatGPT also performed strongly. Gemini, DeepSeek, and LLaMA were rated lower and did not show significant differences among themselves. The high level of international participation and the broad range of clinical experience among respondents enhance the generalizability of these findings. Despite the superior performance of certain models, nearly half of the surveyed surgeons expressed only moderate confidence (“may be”) in recommending AI language models to patients for medical information. This highlights ongoing concerns about the reliability and trustworthiness of AI-generated content in healthcare. Overall, while AI language models, particularly Grok and ChatGPT, show promise as supplementary tools for patient education, further improvements and validation are needed before they can be fully endorsed for independent use in clinical practice.

AI is revolutionizing healthcare by making medical information more accessible to the public. However, ensuring the reliability of AI-generated responses is critical, particularly for medical procedures like RFA. By systematically evaluating and ranking AI responses through expert assessment, this study provides valuable insights into the capabilities of AI in patient education. Our findings will help guide both patients and healthcare providers in making informed decisions about utilizing AI as a trusted medical information source. As AI continues to evolve, ongoing research like this will be essential to ensure that its application in healthcare is both accurate and beneficial to patient outcomes.

## Appendices

**Table 8:** FAQ’s to assess AI accuracy in patient education regarding RFA for varicose veins We asked the respondents to evaluate the answers based on: accuracy (correctness of medical facts); clarity (how easily patients can understand the response); relevance (whether the response directly answers the question); and depth (level of detail provided). FAQ: frequently asked questions, RFA: radiofrequency ablation Credits: Ayman Zyada, Ayman Fakhry

Question No.	Questions
Q1	What is radiofrequency ablation of varicose veins, and how does it work?
Q2	What potential risks and complications are associated with the procedure?
Q3	What do I need to do before the procedure?
Q4	How long does the procedure take?
Q5	Is it painful? Will I be under general anesthesia?
Q6	After the procedure, can I drive, practise exercise, air travel?
Q7	How long is the recovery period, and when can I return back to work?
Q8	How soon after the procedure will my symptoms improve?
